# Multisystem Inflammatory Syndrome Associated with SARS-CoV-2 Infection in an Adult: A Case Report from the Maldives

**DOI:** 10.3390/tropicalmed6040187

**Published:** 2021-10-19

**Authors:** Ahmed Miqdhaadh, Hisham Ahmed Imad, Aminath Fazeena, Thundon Ngamprasertchai, Wang Nguitragool, Emi E. Nakayama, Tatsuo Shioda

**Affiliations:** 1 Department of Medicine, Indira Gandhi Memorial Hospital, Malé 20002, Maldives; migu313@hotmail.com (A.M.); aminathfazyna@gmail.com (A.F.); 2 Mahidol Vivax Research Unit, Faculty of Tropical Medicine, Mahidol University, Bangkok 10400, Thailand; wang.ngu@mahidol.edu; 3 Department of Viral Infections, Research Institute for Microbial Diseases, Osaka University, Osaka 565-0871, Japan; emien@biken.osaka-u.ac.jp (E.E.N.); shioda@biken.osaka-u.ac.jp (T.S.); 4 Department of Clinical Tropical Medicine, Faculty of Tropical Medicine, Mahidol University, Bangkok 10400, Thailand; thundon.ngm@mahidol.ac.th; 5 Department of Molecular Tropical Medicine and Genetics, Faculty of Tropical Medicine, Mahidol University, Bangkok 10400, Thailand

**Keywords:** multisystem inflammatory syndrome, adults, clinical manifestations, COVID-19, Maldives

## Abstract

The multisystem inflammatory syndrome in adults (MIS-A) is a novel syndrome observed during COVID-19 outbreaks. This hyper-inflammatory syndrome is seen predominantly in children and adolescents. The case of an adult from the Maldives who had asymptomatic SARS-CoV-2 infection three weeks before presenting to the hospital with fever, rash, and shock is presented. De-identified clinical data were retrospectively collected to summarize the clinical progression and treatment during hospitalization and the six-month follow-up. SARS-CoV-2 infection was confirmed by RT-PCR. Other laboratory findings included anemia (hemoglobin: 9.8 g/dL), leukocytosis (leukocytes: 20,900/µL), neutrophilia (neutrophils: 18,580/µL) and lymphopenia (lymphocytes: 5067/µL), and elevated inflammatory markers, including C-reactive protein (34.8 mg/dL) and ferritin (2716.0 ng/dL). The electrocardiogram had low-voltage complexes, and the echocardiogram showed hypokinesia, ventricular dysfunction, and a pericardial effusion suggestive of myocardial dysfunction compromising hemodynamics and causing circulatory shock. These findings fulfilled the diagnostic criteria of MIS-A. The case was managed in the intensive care unit and required non-invasive positive pressure ventilation, inotropes, and steroids. With the new surges of COVID-19 cases, more cases of MIS-A that require the management of organ failure and long-term follow-up to recovery are anticipated. Clinicians should therefore be vigilant in identifying cases of MIS-A during the pandemic.

## 1. Introduction

The multisystem inflammatory syndrome in children (MIS-C) is a rare and distinct complication occurring predominantly in boys 6–10 years of age and is associated with the coronavirus disease-19 (COVID-19) outbreak [1,2]. This syndrome, when found in association with severe acute respiratory syndrome coronavirus 2 (SARS-CoV-2) infection, is characterized by a hyper-inflammatory state within the host. The clinical hallmarks of this syndrome include the elevation of inflammatory markers such as the C-reactive protein (CRP) and the acute-phase reactant ferritin, which is normally observed in the presence of organ dysfunction. This syndrome resembles Kawasaki disease, which, without treatment, can lead to complications with increased severity and morbidity resulting from coronary artery dilation and aneurysms [3].

During the first quarter of 2020, similar observations of a hyper-inflammatory syndrome associated with SARS-CoV-2 were reported in adolescents and adults [4,5,6,7,8,9,10,11]. The immunological host response to SARS-CoV-2 can lead to different recognizable immunological phenotypes, including the cytokine release syndrome (CRS) and the MIS-C or the multisystem inflammatory syndrome in adults (MIS-A). In CRS, a downregulated type 1 immune response affects the pulmonary system, leading to pulmonary consolidation, edema, and eventually, acute respiratory distress syndrome [12]. In children and adults, the MIS causes the expression of immunologic mediators, such as cytokines, that have profound impacts on extra-pulmonary organs such as the gastrointestinal tract, the heart, the skin, and the brain. The incidence of MIS-C in children and adolescents was previously reported to be 5.1 persons per 1,000,000 person-months, whereas the frequency of MIS-A has not been reported [13].

At the time when the early cases were reported, what triggered the overt expression of cytokines by the host cells that would cause the physiological abnormalities exhibited in MIS from COVID-19 was largely unknown. It has been postulated that the immunological response is due to the presence of viral antigens or specific antibodies. In children, MIS-C develops after seroconversion, suggesting that the pathogenetic mechanism is a post-infection antibody-mediated response during convalescence [13]. The time to develop MIS-C after exposure to SARS-CoV-2 is reported to be within five weeks after infection [14]. The existing data regarding MIS-A suggest a similar convalescent response in adults [15,16,17,18]. Nevertheless, reports of a multisystem inflammatory syndrome after vaccination (MIS-V) in individuals who had received immunization after recovering from mild COVID-19, or perhaps non-neutralizing antibodies from other coronaviruses, suggest an alternative pathogenesis involving antibody enhancement that leads to the hyper-inflammatory syndrome [19,20,21].

The Maldives, located in the Indian Ocean, has a population of 551,735 people. At the time of writing, the crude fatality rate from COVID-19 was 0.3%, 15% of the population were confirmed to have COVID-19, and over 70% had received at least one dose of the vaccine against SARS-CoV-2. Several tropical infections are endemic to the islands, and the clinical findings of MIS-A may overlap with symptoms and signs of other tropical diseases. These include the clinical setting of plasma leakage in severe dengue, the vasculitis phenomenon in leptospirosis and rickettsiosis, and the aberrant host immune response to the superantigens in toxic shock syndrome and from hemophagocytic lymphohistiocytosis occurring in many infectious diseases. Despite these challenges, it is important to make the diagnosis and begin treatment promptly. Early recognition, with the aid of inflammatory biomarkers in the presence of organ dysfunction, will lead to early targeted therapy against the inflammatory response that causes organ damage.

The clinical progression and course of illness of an adult case of MIS-A from the Maldives is presented in this paper.

## 2. Materials and Methods

The case described here presented to Indira Gandhi Memorial Hospital in Malé, Maldives, in December 2020. De-identified clinical and laboratory data were reviewed using the patient medical chart, which included information during hospitalization and follow-up visits. SARS-CoV-2 infection was confirmed by RT-PCR (Liferiver, San Diego, CA, USA). Dengue diagnostics included SD Bioline Dengue Duo (Abbott Diagnostics, Yonginsi, Gyenoggi, Korea) and Panbio Dengue IgM/IgG ELISA (Abbott Diagnostics). Hemoculture was performed using an automated culture system (Biomeriuex, Durham, NC, USA). The previously described MIS-A case definition was used [22]. The criteria fulfilled were: severe illness requiring hospitalization in a person aged ≥21 years; a positive test result for current or previous SARS-CoV-2 infection (nucleic acid, antigen, or antibody) during admission or in the previous 12 weeks; severe dysfunction of one or more extra-pulmonary organ systems; laboratory evidence of severe inflammation; and absence of severe respiratory illness. Photographs were provided by the attending physician.

## 3. Case Report

**Figure 1:**
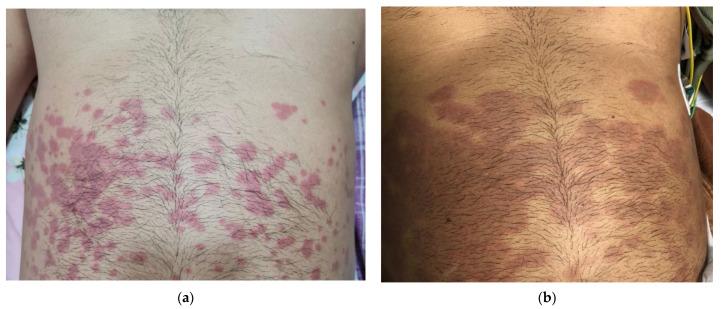
The evolution of a vasculitic rash. (**a**) Progression and clustering of the erupted maculopapular rash on the second day of illness; (**b**) desquamation with coalescence of an erythema multiforme-like lesion on the anterior surface of the abdominal wall upon presentation to the hospital (day six of illness).

A 44-year-old man developed an acute undifferentiated febrile illness with a rash two days after completing 14-day home isolation, as shown in Figure S1. He remained asymptomatic after the confirmation of COVID-19 by RT-PCR. The onset of symptoms was described as mild, intermittent fever associated with chills. A maculopapular rash with lesions of varying sizes (0.5–2 mm) appeared on the abdomen simultaneously with the onset of fever. The rash was non-blanching, non-pruritic, and non-tender to palpation, and it desquamated rapidly into annular plaques with satellite lesions, as shown in Figure 1.

The patient had presented to the emergency room with a complaint of extreme fatigue. There was an additional history of having several episodes of vomiting and diarrhea during the first few days of illness, but he denied experiencing abdominal pain. There was no history of headache, myalgia, arthralgia, bleeding, or any mucosal involvement. In addition, there was no history of any kind of respiratory symptoms, including coryza, sore throat, or cough. The chest X-ray at presentation is shown in Figure S2 and the ECG in Figure S3. Further history included a visit to a private clinic on the third day of illness, resulting in empiric antibiotic treatment (ciprofloxacin 1000 mg/day).

On examination, the patient was conscious and lucid despite being in shock. The blood pressure was 84/52 mmHg, and the pulse was feeble and regular at 137 beats/min. The lower extremities were cool to the touch, with undetectable distal peripheral pulses and a prolonged capillary fill time. The oral temperature was 38.2 °C, and the respiratory rate was 20 breaths/min, with an oxygen saturation of 99–100% in room air. The tachycardia and distant heart sounds were audible with normal breath sounds through auscultation, and there was no neurological deficit. The routine laboratory results showed a left shift with leukocytosis and the predominance of neutrophils and anemia. The inflammatory markers and other biochemical results are shown in Table 1.

The patient received bolus fluid challenges and started receiving prophylactic broad-spectrum antibiotics. Unfortunately, during vigorous resuscitation, the patient developed acute pulmonary edema; the intravenous fluids were consequently discontinued. Inotrope support was started with supplemental oxygen, and the patient was transferred to the intensive care unit (ICU) for further management, including non-invasive positive pressure ventilation. Echocardiography showed global left ventricular hypokinesia with a moderate left ventricular dysfunction. In addition to this, there was mitral and tricuspid valve regurgitation, with the dilation of all four chambers of the heart, pericardial effusion, and an ejection fraction (EF) less than 30%.

After a marked elevation of inflammatory markers in the presence of organ dysfunction, intravenous glucocorticosteroid (hydrocortisone 300 mg/day) was started, given the clinical parameters meeting the MIS-A criteria. Over the next four to six hours, evident clinical improvement was observed. The patient became hemodynamically stable, and the positive pressure ventilator support was discontinued. Unexpectedly, on the third day of admission to the ICU, the patient developed new-onset symptomatic atrial fibrillation with a fast ventricular response, as shown in Figure S4. The abnormal heart rhythm was reverted back to sinus rhythm with antiarrhythmic and rate-controlling agents. A follow-up echocardiography performed prior to discharging the patient from the hospital demonstrated a resolution of the ventricular dysfunction, with an EF of 70%. 

However, residual dilated cardiomyopathy with mild pulmonary arterial hypertension and mild pericardial effusion remained. The patient was discharged on oral prednisolone (30 mg/day), ramipril (1.25 mg/day), metoprolol (100 mg/day), and spironolactone (12.5 mg/day). During the follow-up visits, the medications were tapered and then stopped six months later. The results of the routine investigations performed during the follow-up visits are shown in Table S1.

## 4. Discussion

The pathogenesis of MIS-A may be multifactorial, whereas the clinical trajectory reflects the host inflammatory responses that occur at different clinical phases of COVID-19 [23,24]. This hyper-inflammatory syndrome activates the endothelium, with ensuing thromboinflammation and microvascular dysfunction [25]. Beta coronaviruses show tropism to myocardial endothelial cells, and the cardiovascular system has frequently been affected in MIS-A [22]. The hyperinflammation stuns the heart, triggering myocarditis to cause biventricular failure and conduction defects [22,26]. The pathophysiology of myocarditis entails direct injury to myocytes, after which remodeling occurs, with persistent inflammation resulting in dilated cardiomyopathy [27].

Similarly, in the present patient, there was significant cardiovascular system involvement leading to structural abnormalities and a cardiovascular dysfunction consistent with previously described findings [22]. Part of the treatment included the early administration of an angiotensin-converting enzyme inhibitor, which, in pre-clinical trials, had been shown to reduce the remodeling from myocarditis [28]. Fortunately, the patient had a remarkable recovery and was completely asymptomatic with normal echocardiographic findings and biomarkers not suggestive of any further inflammation at six months. Cardiac magnetic resonance imaging is a valuable tool for detecting active myocardial inflammation, but it is not widely available. Therefore, the subsequent follow-up will be in six months, and if he remains asymptomatic, he will be followed up yearly.

Although the present patient had no past medical history and was otherwise considered a healthy adult, he was overweight (BMI 26.3 kg/m^2^), and obesity has been associated with severe COVID-19 [29,30]. In addition, obesity also contributes to increased host meta-inflammation, and the adipocytes are known to express inflammatory cytokines such as interleukin 6, tumor necrosis factor-alpha, and acute phase reactants such as CRP [31]. As a result, there is sequestration of lymphocytes and other mononuclear cells in adipose tissue. Most of the cutaneous lesions were restricted to the truncal region with maximum adiposity, resembling an erythema multiforme-like rash [32]. A similar, annular rash has been previously described in a patient with MIS-A and heart failure [33]. Histopathological examinations of skin lesions in children have shown perivascular inflammation with the infiltration of lymphocytes [34,35].

Several tropical diseases are endemic to the Maldives, and clinicians use point-of-care test kits since many tropical diseases are indistinguishable upon presentation and can overlap with symptoms of MIS-A [36,37,38]. In the present case, the patient had a positive diagnosis of dengue IgM from ELISA, but this finding had to be carefully interpreted as false-positive dengue serology had been described in COVID-19 patients [39]. Cross-reacting immunoglobulins with other flaviviruses have also been reported, but they could not be demonstrated in this case [40]. Despite the ELISA positive test results for dengue IgM in this case, the rapid test kit results for dengue IgM, IgG, and NS1 were all negative. Together with the clinical findings upon presentation, such as (a) the evidence of the extravasation of fluid into extra interstitial spaces (i.e., pericardial effusion) and (b) the absence of hemoconcentration or a narrowed pulse pressure characteristic of dengue hemorrhagic fever, the etiology of the illness is deemed unlikely to be dengue [41].

The patient denied any travel history and was mainly restricted to his home during the lockdowns. In addition to this, there was no contact with animals (rodents) or any exposure to floods that would indicate possible etiological agents of rickettsiosis and leptospirosis. Non-infectious differential diagnoses to be considered would be drug reaction, eosinophilia, and systemic symptoms (DRESS) [42]. However, the rash appeared before using antibiotics, and he did not meet any of the criteria for DRESS.

There are several limitations in this report. First, due to its retrospective nature, there was no access to any serum from the acute phase or during convalescence from leftover routine investigations to enable the isolation of the virus, demonstrate the expression of inflammatory cytokines, or detect antibodies against SARS-CoV-2 and exclude other likely viruses which are known to cause myocarditis. Furthermore, the cycle threshold (Ct) value from the RT-PCR was only available for the initial confirmation of COVID-19, and it was not possible to trace the Ct value for the second positive test result performed during hospitalization. Lastly, no biopsy was performed to demonstrate the histological changes observed during MIS-A.

SARS-CoV-2 is a respiratory virus that primarily causes upper respiratory tract infections and pneumonia leading to lung fibrosis [43]. Growing evidence suggests the transmission route of the virus to be by aerosol spread, making it a potent contagion [44]. It is possible that SARS-CoV-2 infection can lead to a hyper-inflammatory state in adults, such as the MIS-A described in this report. In conclusion, a severe hyper-inflammatory syndrome with a febrile rash can occur in asymptomatic COVID-19 cases two weeks after a positive test.

## Figures and Tables

**Table 1:** Serial hematological, biochemical profile and inflammatory markers.

Day of Illness	3	4	6	7	8	9	11	12	14
Hospitalization day			1	2	3	4	6	7	9
Leukocyte/µL	17,000	15,200	20,900	21,200	23,400	27,100	22,490	16,833	10,800
Neutrophils/µL	13,815	13,710	18,580	17,935	20,779	23,469	5376	4764	9104
Lymphocytes	1670	5321	5067	4822	5328	5542	258	270	691
Monocytes/µL	1879	188	250	193	300	403	4	0	421
Eosinophils/µL	17	0	0	0	5	31	0	0	
Basophils/µL	17	0	0	0	0	1	0	0	
Platelets/µL	157,000	161,000	207,000	257,000	324,000	413,000	537,000	549,000	499,000
Hemoglobin (g/dL)	12.6	11.3	9.8	9.5	9.1	9.7	10.4	10.6	11.6
Hematocrit (%)	40.1	36.1	31.7	31.4	29.2	30.3	33.7	34.6	36.6
Total bilirubin (mg/dL)		2.0	2.0	1.3	0.8	0.8	0.9	1.0	0.9
Total protein (g/dL)		6.4	6.4	5.3	5.3	5.6	5.1	4.9	5.1
Alkaline phosphatase (IU/L)		38	52	38	43	40	34	37	34
Aspartate aminotransferase (IU/L)		44	51	94	81	19	75	72	75
Alanine aminotransferase (IU/L)		24	31	58	75	94	92	118	20
Creatinine (mg/dL)		0.9	1.0	0.8	0.8	0.8	0.7	0.7	0.8
Urea (mg/dL)		16.9	28.4	37.8	37.8	38.5	32.1	39.5	15.0
CRP (mg/dL)		22.6	34.8	31.7	15.7	8.8	2.6	1.7	0.9
Ferritin (ng/mL)			2716	4235		3499	2453	1840	1949
Fibrinogen (mg/dL)			937						
APTT (sec)			49.5	51.9		33.7			
PT (sec)			16.5	17.2		16.0			
INR			1.5	1.6		1.5			
LDH (IU/L)			199			261			
D-dimer (ng/mL)			1.3						
Troponin I (ng/mL)			1.0	0.6	0.8				
CK (IU/L)			138	101	83				
CK-MB (IU/L)			14	13	33				
RT-PCR SARS-CoV-2			Positive						Negative
Dengue NS1	Negative		Negative						
Dengue IgM	Positive		Positive						Negative
Dengue IgG	Negative		Negative						Negative
Hemo-culture									Negative
Sputum culture									Negative
Urine culture									Negative

CRP: C-reactive protein; APTT: activated partial thromboplastin time; PT: prothrombin time; INR: international normalized ratio; LDH: lactate dehydrogenase; D-dimer: domain dimer; CK: creatinine kinase; CK-MB: creatinine kinase myocardial band; NS1: nonstructural protein 1; IgM: immunoglobulin M; IgG: immunoglobulin G.

## Data Availability

The data presented in this study are available on request from the corresponding author. The data are not publicly available to ensure the privacy of the study participant.

## Supplementary Materials

The following are available online at https://www.mdpi.com/article/10.3390/tropicalmed6040187/s1. Figure S1: Timeline showing the time from exposure to SARS-CoV-2 to developing multisystem inflammatory syndrome (MIS-A); Figure S2: Chest X-ray at the time of presentation to the hospital; Figure S3: Electrocardiogram taken in the emergency room; Figure S4: Clinical course from the time of admission to the intensive care unit to discharge from the hospital; Table S1: Follow-up routine laboratory results.
