# Supplementary material for: Multisystem Inflammatory Syndrome Associated with SARS-CoV-2 Infection in an Adult: A Case Report from the Maldives
Source: Trop Med Infect Dis. 2021 Oct 19;6(4):187. doi: 10.3390/tropicalmed6040187 (PMC8544693; doi:10.3390/tropicalmed6040187)
Supplement: Supplementary file 1 [file tropicalmed-06-00187-s001.zip › tropicalmed-1397194-supplementary.pdf]

Tropical Medicine and Infectious Disease

MDPI

Supplementary Materials

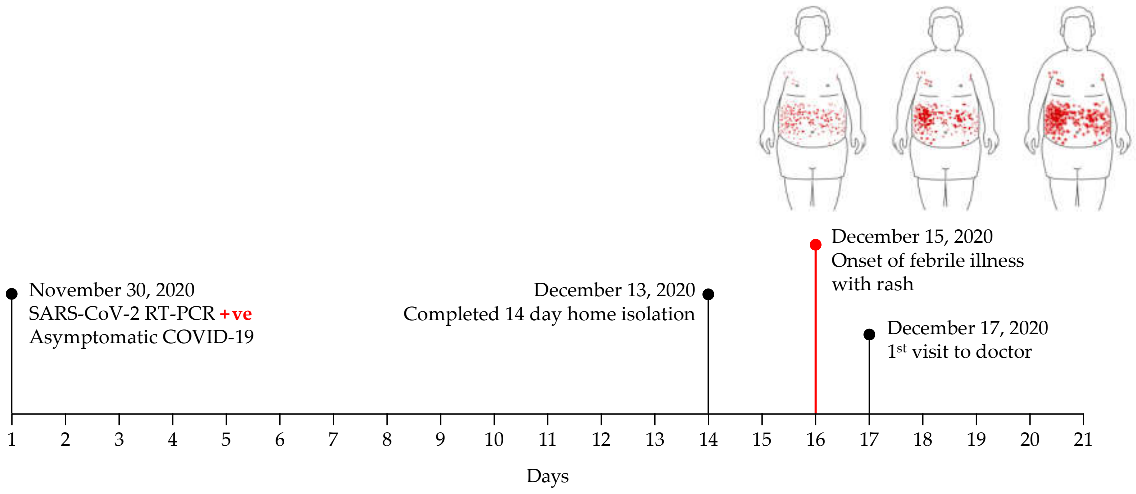

Figure S1. Timeline showing the time from exposure to SARS-CoV-2 to the development of multisystem inflammatory syndrome (MIS-A).

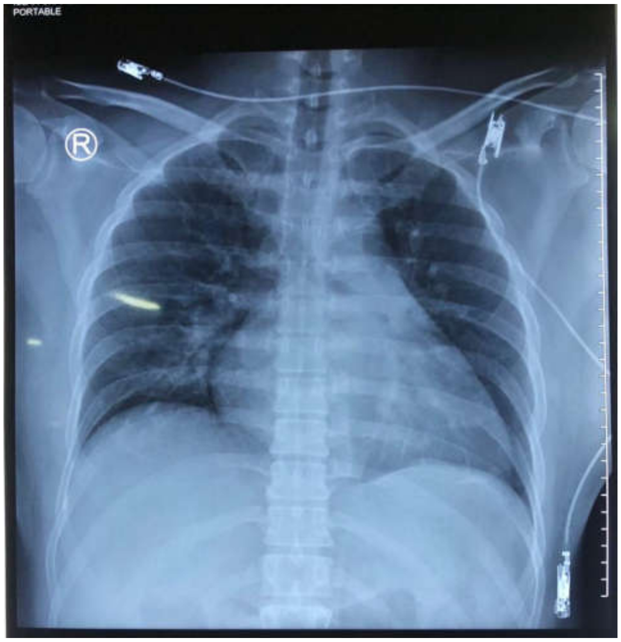

Figure S2. Posterior-anterior view of the chest X-ray at the time of presentation to the hospital. There is an increased cardiac silhouette seen with clear cardio-phrenic and costo-phrenic angles and clear lung fields.

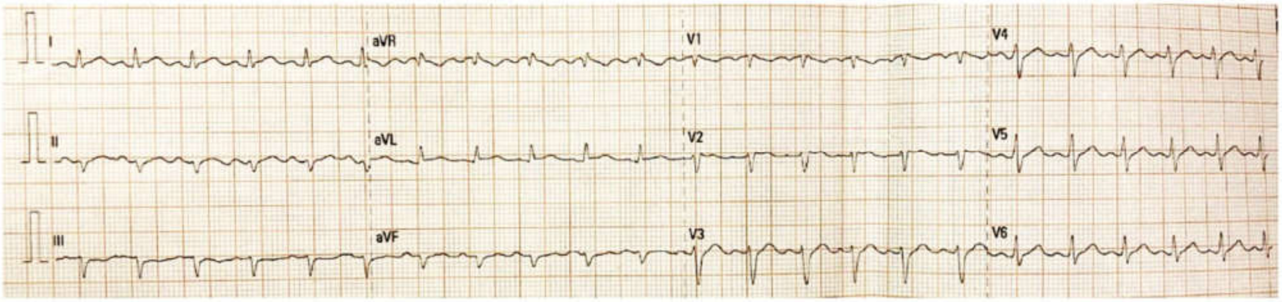

Figure S3. Electrocardiogram taken in the emergency room on the day of presentation to the hospital. The ECG shows low-voltage wave complexes, sinus tachycardia with left axis deviation and poor progression of the R wave, including non-specific ST-T wave changes.

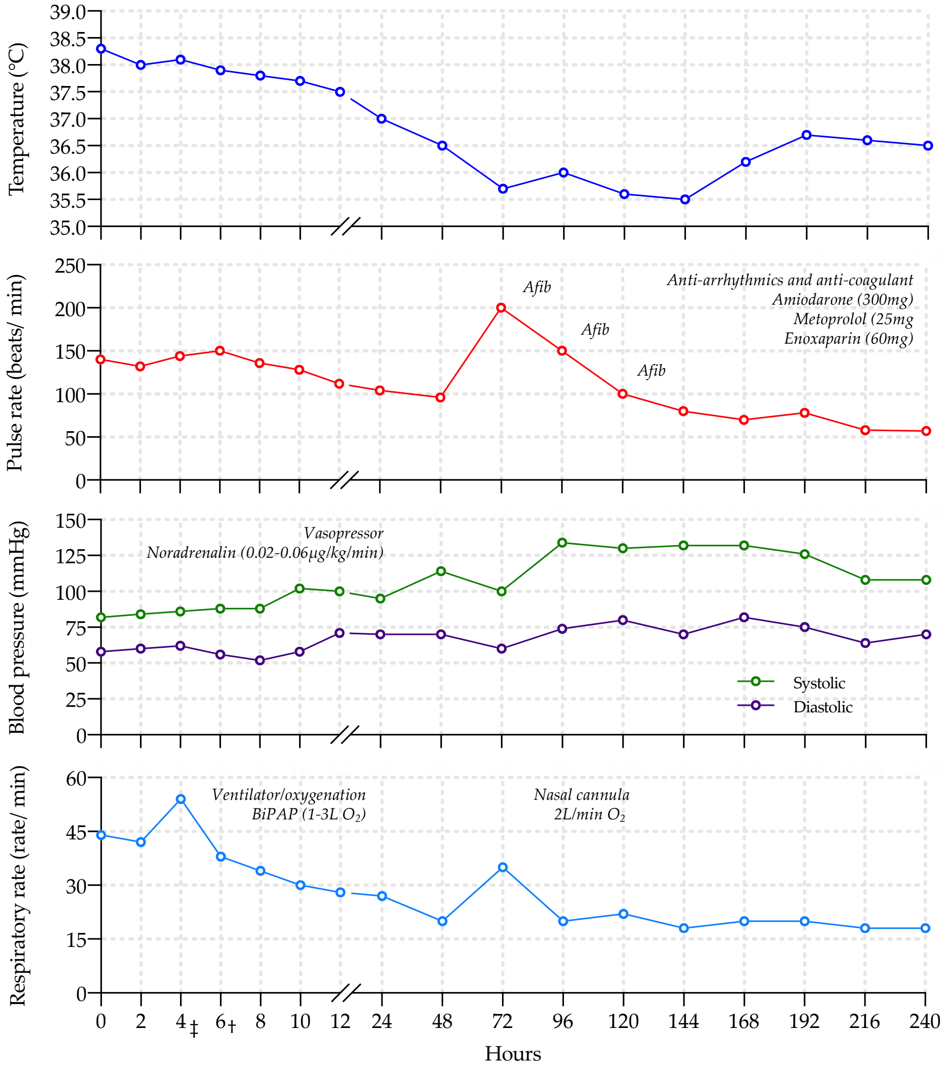

Figure S4. Clinical course from the time of admission to the intensive care unit to discharge from the hospital. The shaded areas in blue, green, and red represent the period's prompt interventions that were required to stabilize the hemodynamics of the patient. BiPAP: bi-level positive airway pressure; Afib: atrial fibrillation; ‡ Diuretic (Furosemide 80 mg) was administered; † Glucocorticosteroid (hydrocortisone 300 mg/day) was started.

Table S1. Follow-up routine laboratory results

**Follow-up Date**	**07/01/21**	**23/01/21**	**17/02/21**	**14/4/21**
Leukocyte/μL	10,500	5,870	9,040	10,500
Neutrophils/μL	6948	3686	4113	7371
Lymphocytes	738	986	4040	2257
Monocytes/μL	141	1027	687	714
Eosinophils/μL	15	158	63	10
Basophils/μL	23	11	81	63
Platelets/μL	301,000	182,000	217,000	291,000
Hemoglobin (g/dL)	13.9	12.8	14.0	14.5
Hematocrit (%)	45.7	43.4	48.9	48.8
Bilirubin (mg/dL)	0.70	1.20		
Total protein (g/dL)	6.70	6.20		
Alkaline phosphatase (IU/L)	54	68		25
Aspartate aminotransferase (IU/L)	40	63		22
Alanine aminotransferase (IU/L)	119	73		25
Creatinine (mg/dL)	0.95	0.72	0.88	1.12
Urea (mg/dL)	34.2	14.5	19.2	
CRP (mg/dL)	0.08	6.39		0.12
Ferritin (ng/mL)	839		256	211
Fibrinogen (mg/dL)			296	259
Troponin I (ng/mL)			0.0	0.0
CK (IU/L)		32	59	44
CK-MB (IU/L)		18	16	21

CRP: C-reactive protein; LDH: lactate dehydrogenase; CK: creatinine kinase; CK-MB: creatinine kinase myocardial band
